# PRP coating on different modified surfaces promoting the osteointegration of polyetheretherketone implant

**DOI:** 10.3389/fbioe.2023.1283526

**Published:** 2023-11-03

**Authors:** Xiaotong Shi, Zongliang Wang, Min Guo, Yu Wang, Zhiguo Bi, Dongsong Li, Peibiao Zhang, Jianguo Liu

**Affiliations:** ^1^ Department of Orthopedic Surgery, The First Hospital of Jilin Uniersity, Changchun, China; ^2^ Key Laboratory of Polymer Ecomaterials, Changchun Institute of Applied Chemistry, Chinese Academy of Sciences, Changchun, China

**Keywords:** polyetheretherketone, surface treatment, platelet-rich plasma, growth factor, osteointegration

## Abstract

**Introduction:** Polyetheretherketone (PEEK) material implants have been applied more and more clinically recently. In order to increase the osteogenic activity of PEEK material, the microstructure change of the material surface and the construction of functional microcoatings have become a hot research topic. This study investigated the ability of PEEK surfaces modified by different methods to carry Platelet-rich plasma (PRP) and the osteogenic ability of different PEEK microstructures after carrying PRP *in vivo*/*in vitro*.

**Methods:** In this study, PEEK surfaces were modified by sulfuric acid, gaseous sulfur trioxide and sandpaper. Next, PRP from SD rats was prepared and incubated on PEEK material with different surface microstructures. Lactate dehydrogenase test, scanning electron microscope and Elisa assay was used to evaluate adhesion efficiency of PRP. Then *in vitro* tests such as CCK-8, ALP staining, ARS staining and RT-qPCR et al were used to further evaluate osteogenesis ability of the PRP coating on PEEK surface. Finally, The tibia defects of SD rats were established, and the new bone was evaluated by Micro-CT, HE staining, and immunofluorescence staining.

**Results:** The sandpaper-polished PEEK with the strongest PRP carrying capacity showed the best osteogenesis. Our study found that the modified PEEK surface with PRP coating has excellent osteogenic ability and provided the basis for the interface selection of PRP for the further application of PEEK materials.

**Discussion:** Among the three PEEK modified surfaces, due to the most PRP carrying and the strongest osteogenic ability *in vitro/vivo*, the frosted surface was considered to be the most suitable surface for the preparation of PRP coating.

## 1 Introduction

Bone defects caused by various reasons, such as tumor surgery and trauma, are common worldwide. Therefore, bone defect repair and osseointegration of bone replacement materials have been a hot topic worldwide (Quarto and Giannoni, 2016). At present, titanium and its alloy are the most used bone implant materials in clinical due to their good mechanical properties, chemical stability, and biocompatibility. However, the persistent release of metal ions, anaphylaxis, and stress shielding are its side effects difficult to solve (HuiskesWeinans and Van, 1992; Niki et al., 2001).

Therefore, in recent years, in order to overcome the shortcomings of titanium alloys, PEEK with an elastic modulus closer to human bone tissue has become a powerful alternative to titanium (Lee et al., 2012; Ruiter et al., 2021; Vogel et al., 2021). However, the biological inertness of PEEK materials has hindered its clinical application. There are three kind methods to improve the biological activity of PEEK material: First, change the surface morphology of PEEK material to facilitate cell adhesion, growth and differentiation; Second, the PEEK material is blended with other bioactive materials; Third, bioactive factors were grafted on the PEEK surface by chemical and physical methods (Vogel et al., 2021). Previous studies have shown that there are a variety of ways to change the surface shape of PEEK materials. The surface morphology of PEEK with different roughnesses can be obtained by grinding the PEEK surface with sandpaper of different roughnesses (Sunarso et al., 2018). Concentrated sulfuric acid can create a three-dimensional pore structure on the surface of PEEK, enhance the osteogenesis of PEEK, and give PEEK material a certain drug loading ability (Wu et al., 2018; Zhu et al., 2019; Ma et al., 2020). In previous studies in our laboratory, gaseous sulfur trioxide was used to fabricate hydrophilic PEEK surfaces with the 3D pore structure. This PEEK modified surface was also proved to have good mineralization and osteogenesis ability *in vitro* (Wan et al., 2020).

Platelet-rich plasma (PRP) is a plasma rich in high concentrations of platelets obtained from animal or human whole blood by centrifugation (Ehrenfest et al., 2009). The number of platelets in PRP is usually more than three times higher than that in whole blood. Platelets in PRP can secrete a variety of growth factors such as transforming growth factor (TGF), vascular endothelial growth factor (VEGF), and platelet-derived growth factor (PDGF) after activation (Ehrenfest et al., 2009). At present, PRP has been widely used to enhance the biological activity of biomaterials such as hydrogels and gelatin. The study showed that the 3D-printed PRP-GelMA hydrogel prepared by Jiang et al. could promote cartilage regeneration through macrophage immunomodulation (Jiang et al., 2021). Naga et al. demonstrated that perfusion of PRP into 3D microvasculature promoted endothelial cell maturation and improved cell function within 24 h (Nagao et al., 2019). In addition, injectable PRP has been widely used in orthopedic surgeries such as spinal fusion and joint replacement due to its excellent osteopromoting ability (Zavadil et al., 2007; Imagama et al., 2017). However, few studies have reported PRP as a bioactive factor to improve the osteogenic activity of PEEK materials. Therefore, we intend to develop a new method using PRP to improve the biological activity of PEEK materials. At the same time, we believe that morphology is an important factor affecting the biological activity of PRP on PEEK surface.

**SCHEME 1:**
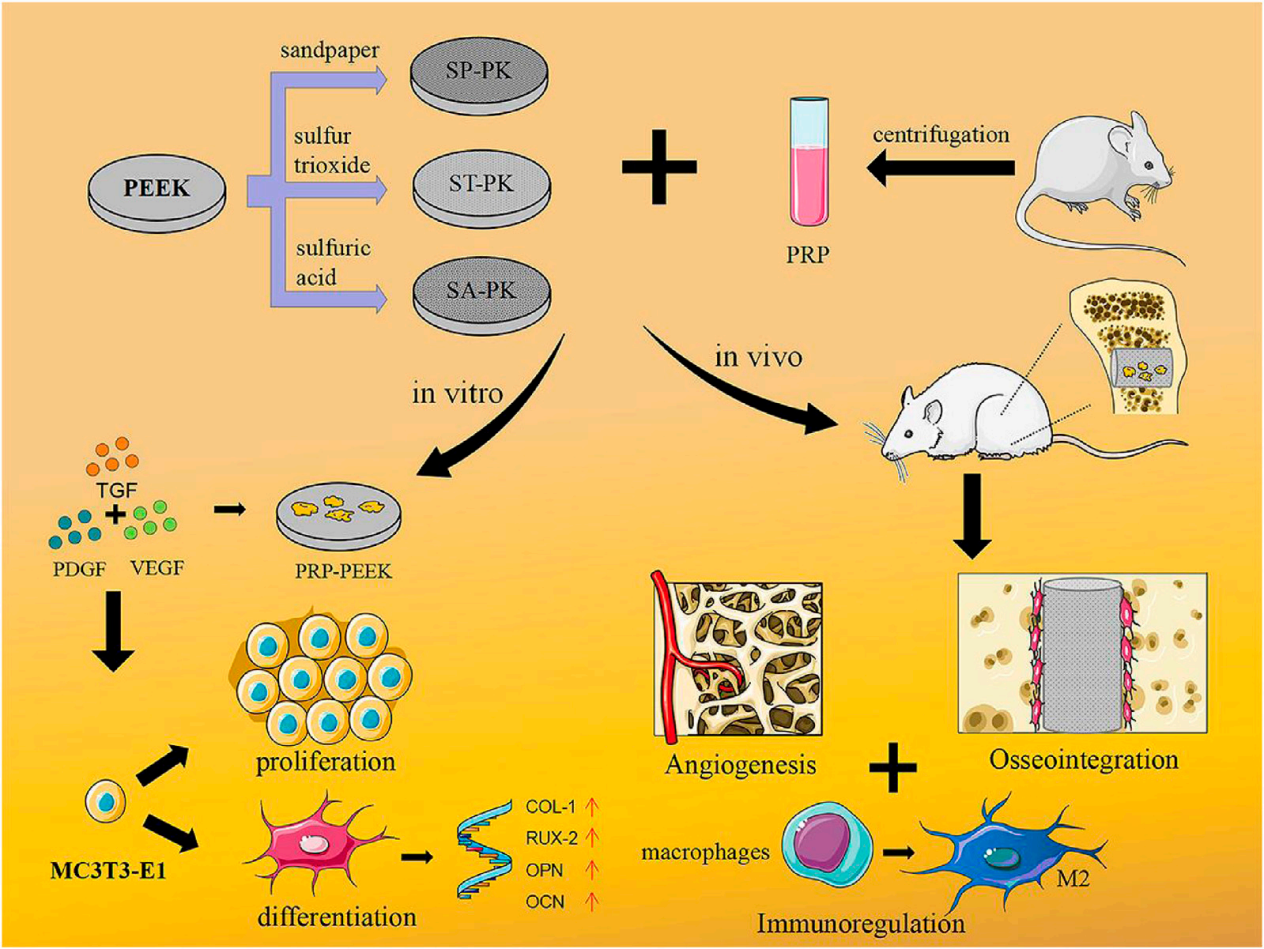
Concept illustration. PEEK samples with three different modified surfaces were fabricated and PRP coatings were prepared. This combination treatment promoted osteoblast proliferation and differentiation *in vitro*, while *in vivo* it could regulate immunity, promote angiogenesis, and ultimately promote osseointegration.

Therefore, in this study, we fabricated PEEK surfaces with different microstructures using sandpaper, sulfuric acid, and gaseous sulfur trioxide. Firstly, we used SEM, EDS, AFM, and universal testing machines to compare and analyze the different morphologies and material characteristics of PEEK. Secondly, we used SEM, LDH test and Elisa to analyze the adhesion characteristics and growth factor release of PRP on PEEK surfaces with different morphologies. Finally, *in vivo* and *in vitro* experiments, we compared and analyzed the osteogenic ability of PEEK materials with different morphologies equipped with PRP (Scheme 1).

## 2 Materials and methods

### 2.1 Preparation of PEEK surface

Biograde PEEK (Victrex, United Kingdom) was fabricated as disks (diameter 15 mm, thickness 0.5 mm) for physicochemical characterization and *in vitro* cell experiments, and cylinders (diameter 2 mm, height 3 mm) for mechanical tests and *in vivo* studies. These samples were ultrasonically rinsed in acetone, absolute ethanol, and deionized water for 30 min and then dried under vacuum to give the original PEEK (named PK).

The original PEEK was fully polished with 220-mesh sandpaper to obtain the frosted PEEK (named SP-PK). The sanding direction was in a single direction and the sanding time was 1 min.

According to the previous research of our research group, the reaction of H_2_SO_4_ and P_2_O_5_ was used to produce gaseous sulfur trioxide (SO_3_), and the SO_3_ modified PEEK surface was obtained (named ST-PK) (Wan et al., 2020). Briefly, PEEK disks were placed on the bracket of refitted glassware and P_2_O_5_ was placed at the bottom. H_2_SO_4_ was then titrated into glassware through a constant voltage funnel. The whole apparatus was maintained in a 75°C thermostatic water bath to maintain the gaseous state of SO_3_, and the exposure time of the PEEK disks to SO_3_ was set to 60 min. Finally, these samples were also ultrasonically rinsed in acetone, absolute ethanol, and deionized water for 30 min and then dried under vacuum.

According to previous studies, PEEK disks were placed in concentrated sulfuric acid to obtain sulfonated PEEK surface (named SA-PK). In brief, the PEEK disks were immersed in concentrated sulfuric acid (95–98 wt%) with ultrasonic stirring for 1 min at room temperature. Finally these samples were ultrasonically cleaned and vacuum dried as described above.

### 2.2 Characterization of PEEK surface

To observe the morphological attributes of the PEEK surface, dried PEEK disks were evaluated with a scanning electron microscope (XL-30 ESEM FEG Scanning Electron Microscope; FEI Company, Hillsboro, OR, United States) after being sprayed with gold. The surface elemental distribution of the PEEK disks was analyzed using energy-dispersive X-ray spectroscopy (EDS). The hydrophilicity/hydrophobicity of these sample surfaces were evaluated by water contact angle measurements (VCA 2000; AST Products Inc., United States): 2 lL DI water droplets were dropped onto the PEEK sample surfaces at room temperature; pictures were taken by a camera after stabilization. Atomic force microscopy (AFM, Veeco, United States) was used to detect the surface structure of modified PEEK surface. Fourier transform infrared spectroscopy (FT-IR; PerkinElmer, FT-IR-2000) was used to measure the chemical structure in the wavenumber region from 400 to 3,500 cm^-1^.

Since PEEK material implants mainly bear compressive stress *in vivo*, we tested the effects of three different surface modification methods on the compressive mechanical properties of PEEK material according to the National Standard of China (GB/T1039). At room temperature, the compressive mechanical properties of PEEK cylinders (diameter 2 mm, height 3 mm) with various modified surfaces were measured by a universal mechanical testing machine (Instron 1121, United Kingdom) with a speed of 2 mm/min.

### 2.3 Fabrication of PRP-PEEK surface

According to previous reports, SD rat-derived PRP was prepared (Zavadil et al., 2007). First, 1 mL of anticoagulant was added to 5 mL of whole blood extracted from SD rats. Then, erythrocytes were removed after centrifugation at 2,000 g for 3 min. Finally, the mixture was centrifuged at 5,000 g for 5 min to obtain the supernatant and platelet precipitate. PRP was obtained by resuspension of the platelet precipitate. An automated counter (Sysmex, XS-800i, Kobe, Japan) was used to analyze platelet concentrations in whole blood and PRP.

Sterile PEEK disks (diameter 15 mm, thickness 0.5 mm) were placed in 24-well plates, and 200 μL of PRP solution was added to each well and incubated in a 37°C incubator for 2 h. At the end of the incubation, each PEEK sheet was washed three times with sterile PBS to obtain PEEK disks with PRP coating (named PRP-PK, PRP-SP-PK, PRP-ST-PEEK and PRP-SA-PEEK respectively).

### 2.4 Evaluation of PRP carrying capacity of PEEK surface

#### 2.4.1 Platelet adhesion observation

Platelet adhesion on various modified PEEK surfaces was observed by SEM after incubation with PRP. Before performing SEM observations, PEEK disks with PRP incubated on the surface were transferred to new 24-well plates and fixed with 2.5% glutaraldehyde(in PBS buffer) for 8 h at 4°C. Then they were dehydrated in 30%, 50%, 70%, 90%, and 100% ethanol solution for 30 min and dried under vacuum.

#### 2.4.2 Quantitative analysis of platelet adhesion

The LDH cytotoxicity kit (Beyotime; Shanghai, China) was used to quantitatively analyze the number of platelets adhered to the surface of different modified PEEK discs. The test procedure was carried out according to the instructions and references (Tamada et al., 1995; Grunkemeier et al., 1998). The number of platelets in PRP solution was recorded as A after determination with an automatic hematology analyzer, and the OD value was recorded as B after LDH detection of PRP solution. The OD value of platelets adhered to the PEEK surface after LDH detection was recorded as C, thus the number of platelets adhered to the PEEK surface *n* = A × C/B. LDH assays were performed at least three times for each surface, and the number of adhered platelets obtained was then averaged.

#### 2.4.3 Assessment of PDGF release

An enzyme-linked immunosorbent assay (ELISA) was used to evaluate PDGF-BB release from different PEEK surfaces modified with PRP. PRP-modified PEEK disks were placed into 24-well plates, and after 1 mL sterile PBS solution was added to each well, the plates were incubated at 37°C. The PBS solution was collected and stored at 20°C at preset time points (0, 2, 4, 8, 12, 24 h) and an equal volume of new PBS solution was added to the wells. An ELISA kit (Solarbio; Beijing, China) was used to measure the release of PDGF-BB from modified PEEK discs, and the absorbance was measured at 450 nm (Infifinite M200, Tecan, Switzerland). A standard curve was also plotted to calculate cumulative release of PDGF.

### 2.5 *In vitro* cell-material interactions assay

#### 2.5.1 Cell culture

In this study, MC3T3-E1 cells were used to evaluate the cytocompatibility and osteogenic activity of different PEEK surfaces before and after PRP incubation. In a humidified atmosphere (37°C, 5% CO_2_), the cells were incubated in Dulbecco’s Modified Eagle Medium (DMEM; Gibco, Thermo Fisher Scientifific, United States) containing 10% fetal bovine serum (Gibco, Thermo Fisher Scientific, United States) and 1% penicillin/streptomycin (Sigma-Aldrich, United States). When the cells were >90% confluent, cell culture medium was aspirated and the cells were then trypsinized, centrifuged and resuspended for cell passage. When the cells were passed to the 3 to 5 passages, they were digested down for cell experiments. 2 × 10^4^ cells were seeded onto the PEEK surfaces in a 24-well plate and DMEM was changed every other day.

#### 2.5.2 Cell adhesion

When 2 × 10^4^ cells on the PEEK surfaces were cultured for 24 h, they were rinsed twice with PBS and fixed with 4% Paraformaldehyde (PFA). Nuclei were stained with 4’,6-diami dino-2-phenylindole (DAPI; Sigma-Aldrich, United States) and visualized under fluorescence microscopy (TE 2000U, Nikon, Japan). Nuclei from at least three independent areas of each type of PEEK surface were counted for quantitative analysis.

The cell morphology of MC3T3-E1 grown on PEEK surfaces was visualized by SEM. Briefly, after cells were incubated on the PEEK surface for 24 h, PEEK samples were washed with PBS and fixed with 4% PFA at room temperature. This was followed by dehydration with gradient concentration of ethanol (50%, 60%, 70%, 80%, 90%, and 100%) for 30 min. Finally, SEM observations were performed after gold spraying on dry PEEK surface.

#### 2.5.3 Cell proliferation

Cell proliferation ability on the surface of PEEK was detected by The Cell Counting Kit-8 (CCK-8; Beyotime Institute of Biotechnology, Shanghai, China). After the cells were seeded onto the PEEK surface at predetermined time points, the original cell culture medium in the 24-well plate was discarded and 1 mL of fresh DMEM containing 10%CCK-8 reagent was added. After 4 h of co-culture, the absorbance value was measured at 450 nm (Infifinite M200, Tecan, Switzerland).

#### 2.5.4 Alkaline phosphatase (ALP) staining and quantitative analysis

Cells on the PEEK surfaces were cultured for 7 and 14 days, then ALP staining and quantitative analysis were performed to evaluate early osteogenesis ability. After washing the PEEK disks twice with PBS, they were fixed with 4% PFA for 15 min at room temperature, then washed again and stained with BCIP/NBT (Beyotime; Shanghai, China). Finally, the PEEK surface was observed under microscope and photographed.

The quantitative analysis of ALP was performed using ALP quantification kit (Beyotime; Shanghai, China). After cells were cultured on PEEK surfaces for 7 and 14 days, they were washed three times with PBS, lysis buffer was added and cell lysate supernatants were collected. The supernatant was co-cultured with p-nitrophenyl phosphate at 37°C for 30 min, and the 405 nm absorbance was measured. Meanwhile, the standard curve was drawn according to the instructions of ALP kit, and the ALP activity was calculated according to the standard curve.

#### 2.5.5 Alizarin red S (ARS) staining and quantitative analysis

Alizarin red S (ARS) staining and quantitative analysis were used to assess extracellular matrix calcium deposition at late stages of osteogenic differentiation. Briefly, cells on the PEEK surface were cultured for 21 days, rinsed and fixed as described in 2.5.4. The PEEK surface was then immersed in ARS solution for staining for 30 min 37°C. Finally, the PEEK surfaces were observed under microscope after washing off excess ARS with PBS.

After the observation, the ARS-stained PEEK surface was immersed in a 10% cetylpyridinium chloride (CPC) solution at 37°C for 2 h, and the absorbance was measured at 540 nm at the end.

#### 2.5.6 Real-time quantitative polymerase chain reaction (RT-qPCR) for osteogenesis

RT-qPCR was used to detect the expression of collagen type I (Col-I), runt-related transcription factor 2 (Rux-2), osteopontin (OPN), and osteocalcin (OCN) genes to examine the effect of the PEEK surface on osteogenic differentiation. The primers are listed in Table 1. After 14 days of culture of cells seeded on the PEEK surface, total RNA was extracted with TRIzol reagent (Invitrogen, Carlsbad, CA, United States of America). It was then reverse transcribed into cDNA with an All-In-One 5X RT MasterMix (abm; Vancouver, Canada), and mRNA expression levels were assessed by RT-qPCR with a BlasTaqTM 2X qPCR MasterMix (abm; Vancouver, Canada). Finally, the comparative Ct method (2^−ΔΔCT^) was used to calculate the relative gene expression and normalization was done based on the expression of the endogenous mouse GAPDH gene.

**TABLE 1:** Primers used in RT-qPCR.

Gene	Forward primer	Reverse primer
Col-I	CGCTGGC AA GA ATOGCGATC	ATGCCTCTGTC A CCTTGTTCG
RUNX2	GCC​GGG​AAT​GAT​GAG​AAC​TA	GGACCGTCCACTGTCACTIT
OPN-F	TCA​GGA​CAA​CAA​CGG​AAA​GGG	TCA​GGA​CAA​CAA​CGG​AAA​GGG
OCN-F	AAG​CAG​GAG​GGC​AAT​AAG​GT	TTT​GTA​GGC​GGT​CTT​CAA​GC

### 2.6 *In vivo* bone repair studies

#### 2.6.1 Animals and surgery

In this study, animal experiments were approved by the Laboratory Animal Welfare and Ethics Committee at the Changchun Institute of Applied Chemistry, Chinese Academy of Sciences and were performed in compliance with the National Institutes of Health’s Guide for the Care and Use of Laboratory Animals (NIH Publications No. 8023, revised 1978). A rat model of unilateral proximal tibial defect was used to observe the induction of osteogenesis after the implantation of each component (PK,SP-PK,ST-PK,SA-PK, P-PK,P-SP-PK,P-ST-PK,P-SA-PK). In total, 48 male Sprague-Dawley (SD) rats (10 weeks, 250 ± 15 g) were used for *in vivo* osteogenic experiments, 6 for each group. The rats were operated under general anesthesia with 3% pentobarbital (0.1 mL/100 g), and all procedures were performed under aseptic conditions. First, a hole of 3 mm depth was drilled perpendicular to the long axis of the tibia with a sterile drill bit of 2 mm diameter. Next, the cylindrical PEEK materials of each group were carefully implanted into the bone defect site. Finally, saline was used to clean the surgical site and suture the wound layer by layer. After surgery, rats were injected with penicillin for 3 consecutive days to prevent infection. The rats were euthanized at 4 and 8 weeks after surgery, and tibial specimens were collected and fixed in 4% paraformaldehyde solution for subsequent experiments.

#### 2.6.2 Microcomputed tomography (Micro-CT) evaluation

In this study, micro-CT was used to assess bone regeneration. Collected tibial specimens were scanned with the Skyscan 1172 Micro-CT system (14 mm resolution, 80 kV, nofilter; Bruker Kontich, Belgium) and reconstructed in 3D using multimodal 3D visualization software (Skyscan 1,076 Scanner, Bruker Micro-CT, NV, Kontich, Belgium). Bone volume fraction (BV/TV), trabecular thickness (Tb. Th), trabecular number (Tb. N), and trabecular separation (Tb. Sp) were used as quantitative analysis indicators of bone regeneration, and these indicators were analyzed by the CTAn software (SkyScan, Belgium).

#### 2.6.3 Histological evaluation

Rat tibial specimens obtained at 4 and 8 weeks were fixed in 4% paraformaldehyde, and the PEEK implant was removed after decalcification for 45 days. Then, bone tissue was sectioned by an electric slicer after being embedded in paraffifin. Hematoxylin and eosin (HE) stain and Sirius red stain were used for general analysis of the new bone and collagen evaluation, respectively. Finally, an imaging system (NIKON DS-U3, Japan) was used to observe these sections.

Immunofluorescence staining of OPN and OCN was performed to analyze osteogenesis. Briefly, tibial specimen sections were blocked in serum and incubated with antibodies against the osteogenic marker antibody Col-I at 4°C overnight. Then, the sections were treated with a secondary antibody with immunofluorescence label. Finally, the sections were observed under a fluorescence microscope.

### 2.7 Statistical analysis

All data are presented as mean ± standard deviation (SD). Each experimental condition was independently tested a minimum of three times. SPSS software (IBM, Chicago, United States) was used for all statistical analyses. Differences between experimental groups were statistically analyzed by one-way analysis of variance (ANOVA) and Tukey’s multiple comparison test A *p*-value of <0.05 was considered statistically significant.

## 3 Results

### 3.1 Characterization of PEEK surface

**FIGURE 1:**
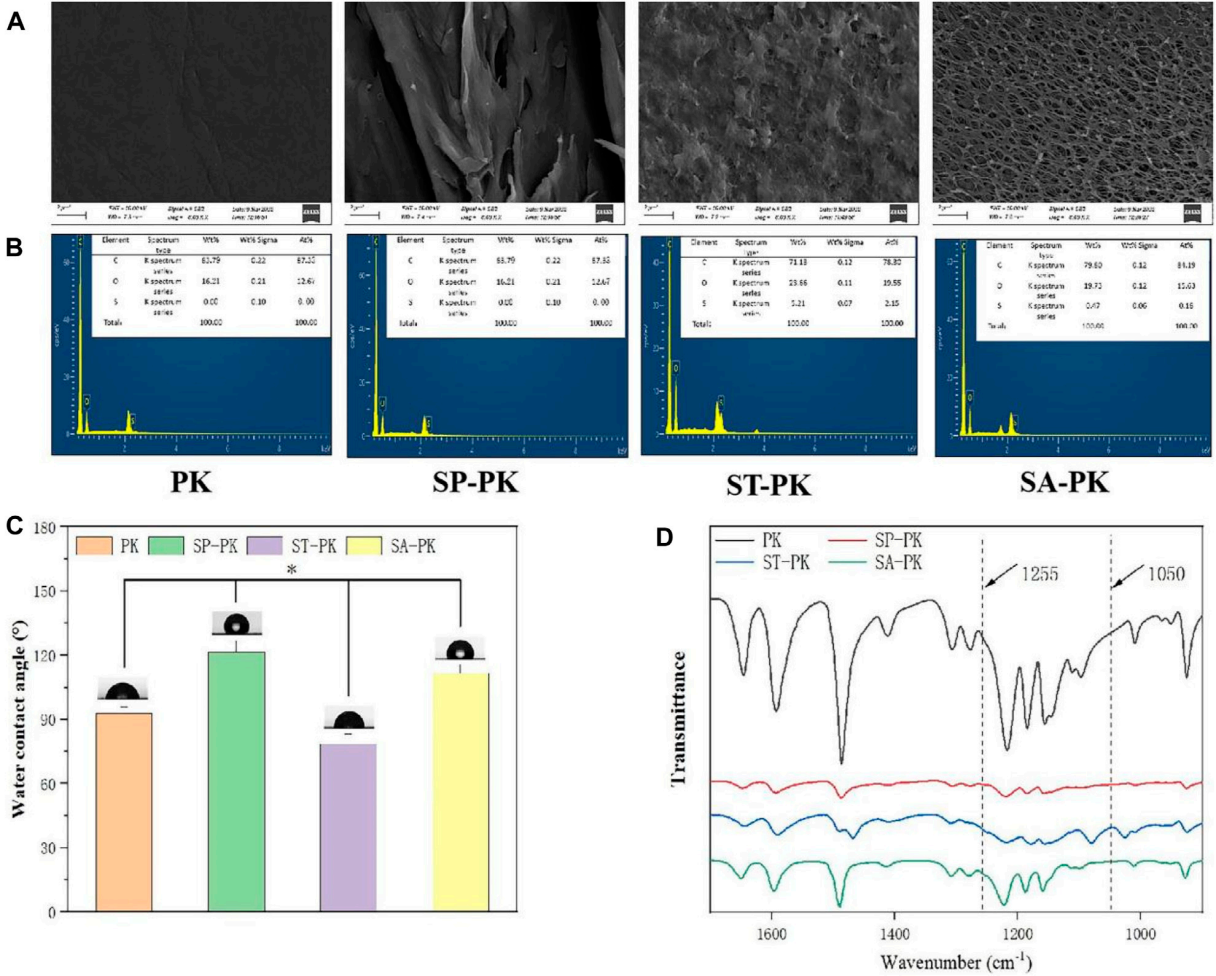
**(A)** The SEM morphology of the surface of PEEK samples from each group. **(B)** EDS results of elemental composition on the surface of PEEK samples in each group. **(C)** Results of water contact angles on PEEK surfaces in each group. **(D) **FT-IR spectral characteristics of PEEK surfaces in each group, *n* = 5.* indicate *p* < 0.05.

**FIGURE 2:**
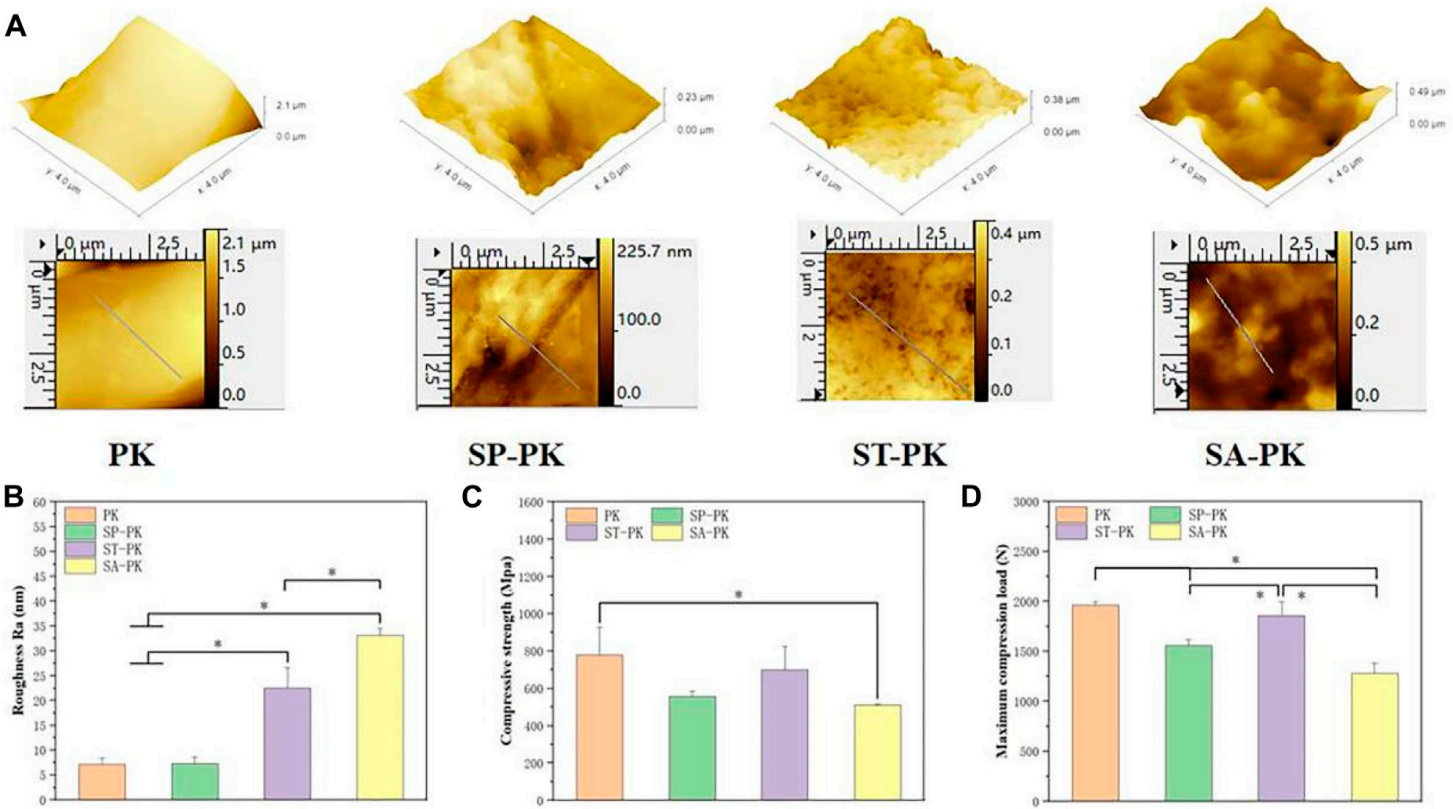
**(A)** AFM images of the surfaces of PEEK samples from each group. Yellow arrows: fully activated platelets; Red arrows: non-activated platelets. **(B)** Surface roughness of each group of PEEK samples analyzed based on AFM images, *n* = 3. **(C)** Compressive strength of PEEK samples in each group, *n* = 5. **(D)** Maximum compression load of PEEK samples in each group, *n* = 5.* indicate *p* < 0.05.

The SEM results revealed the microstructure of four different PEEK surfaces including original PEEK. As shown in Figure 1A, the original PEEK surface is relatively flat; a gully like microstructure was constructed on the surface of the frosted PEEK. The sulfur trioxide treatment formed potholes and surface micropores on PEEK surface. The concentrated sulfuric acid treatment formed a 3D pore structure on the PEEK surface. The nanoscale microstructure and roughness of four PEEK surfaces were measured by AFM (Figure 2A). Consistent with the SEM results, the PK surface was flat and SP-PK surface had gully shape. However, a fine pore structure was observed on the ST-PK surface; coarse and deep crater-like structures were observed on the SA-PK surface. The roughness of different PEEK surfaces was analyzed (Figure 2B). The results showed that there was no significant difference in surface roughness between SP-PK (7.16 ± 1.28 nm) and PK (7.30 ± 1.30 nm) surfaces. The surface roughness of ST-PK group (22.54 ± 4.05 nm) was significantly higher than that of PK and SP groups, and the surface roughness of SA-PK group (33.10 ± 1.33 nm) was significantly higher than that of the other three groups.

The chemical characteristics of the PEEK surfaces of each group were evaluated using FT-IR in the range of 1,700 cm^-1^ to 900 cm^-1^ wavenumber (Figure 1D). In the spectrogram, all characteristic peaks can be identified. The diketobenzene band at positions 1650 cm^-1^,1490 cm^-1^, and 926 cm^-1^;C-O-C stretching vibration of diaryl groups at position 1188 cm^-1^ and 1158 cm^-1^, C = C of the benzene ring in PEEK position at 1600 cm^-1^. According to the results of FT-IR, the waveforms of the SP-PK and SA-PK groups had the same trend as that of the PK group. In the waveform of ST-PK, a peak related to O= S=O at 1255 cm^-1^ and a peak related to S= O at 1050 cm^-1^ could be found. This indicates that the sulfur trioxide gas sufficiently introduces -SO_3_H group to the PEEK surface. The EDS analysis results show the elemental composition of PEEK surface of each group (Figure 1B). Both the ST-PK and SA-PK groups contained S elements-PK group was significantly higher than that in the SA-PK group. Thus, it is also proved that both sulfur trioxide and sulfuric acid introduce -SO_3_H group to the PEEK surface, and the amount of -SO_3_H group introduced by sulfur trioxide is more.

The water contact angle of each PEEK surface was evaluated and the results are shown in Figure 1C. The water contact angle in the PK group was 92.98 ± 2.92°. Among the four groups, the water contact angle of the SP-PK group was significantly higher than that of the other three groups (121.52 ± 5.29°, *p* < 0.05). The water contact angle in the ST-PK group was significantly lower than that in the other three groups (78.64 ± 4.45°, *p* < 0.05). The water contact angle of the SA-PK group (111.72 ± 3.96°) was significantly higher than that of the PK group and significantly lower than that of the SP-PK group.

The compressive mechanical data of the PEEK samples in each group are shown in Figures 2C, D. The compressive strength of the PK group was 777.63 ± 148.63MPa, and that of the other three groups was lower than that of the PK group (558.10 ± 27.19, 700.39 ± 124.20 and 509.40 ± 6.21 respectively). Only the compressive strength of the SA-PK group was significantly lower than that of the PK group. The maximum compressive loads of the PK and ST-PK groups was 1961.67 ± 34.27N and 1857.33 ± 139.15N, and there was no significant difference between them, however, both of them were significantly higher than those of the SP-PK and SA-PK groups. The maximum compressive load of the SP-PK group was 1555.67 ± 61.08, which was significantly lower than that of the PK and the ST groups, but significantly higher than that of the SA-PK group. The maximum compression load in the SA group was1280.33 ± 102.55, which was significantly lower than the other three groups.

### 3.2 Evaluation of PRP carrying capacity of PEEK surface

**FIGURE 3:**
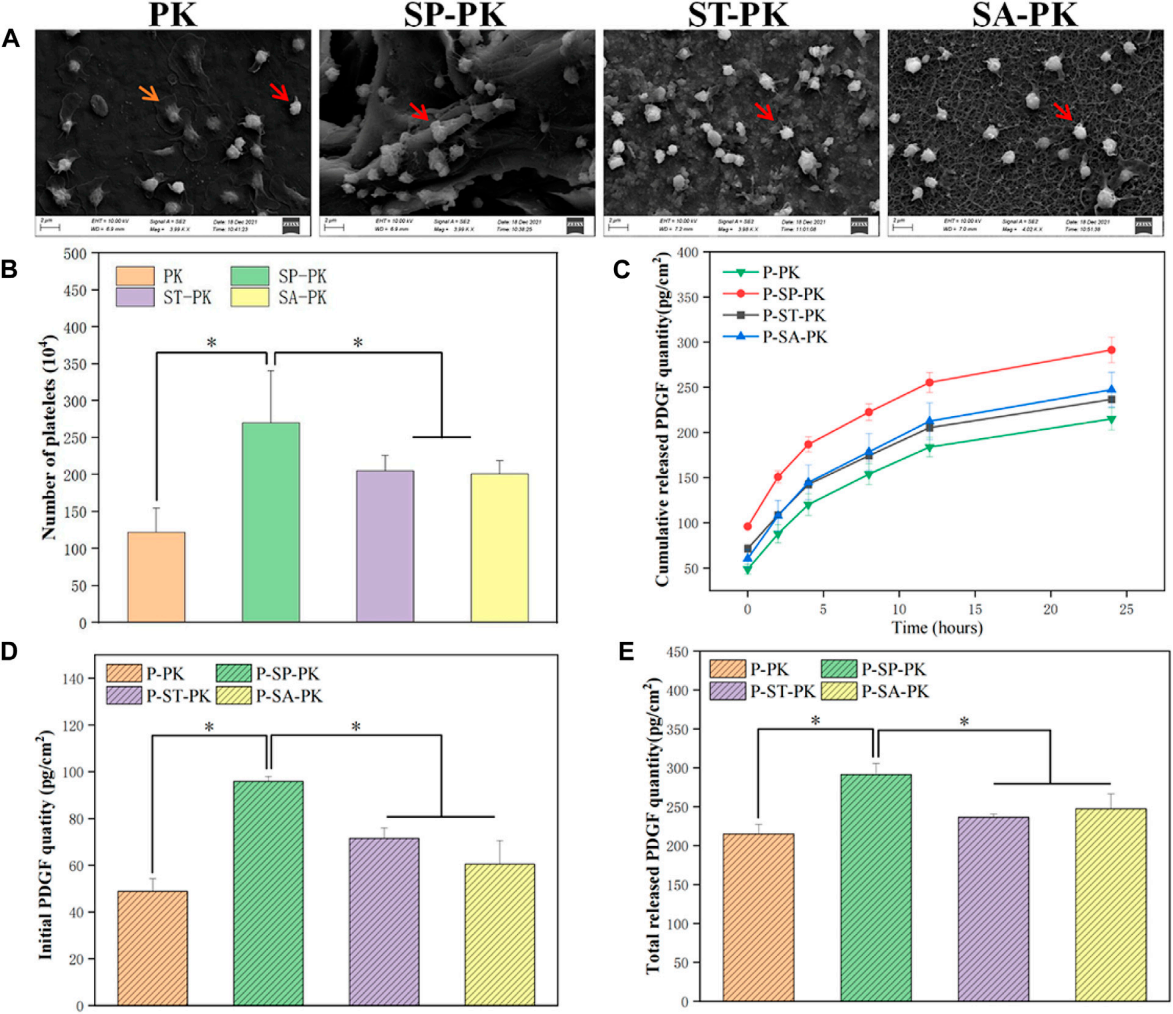
**(A)** SEM results of PEEK samples from each group after incubation with PRP. **(B)** Results of LDH assay for the number of platelets adhered to the surface of PEEK samples in each group, *n* = 3. **(C)** PDGF-BB release curves in each group, n = 3. **(D)** The initial release of PDGF-BB from the surface of each PEEK sample was measured, *n* = 3. **(E)** Final cumulative release of PDGF-BB from the surface of various PEEK samples, *n* = 3.* indicate *p* < 0.05.

The number of platelets in whole blood was 6.74 ± 0.17 × 10^11^, and that in PRP was 34.12 ± 0.21 × 10^11^. The concentration of platelets in PRP was 5.06 times that in whole blood. After incubation of PRP onto the PEEK surfaces of each group, the adhesion of platelets to different PEEK surfaces was observed using SEM (Figure 3A). Platelets that ruptured after full activation are shown by yellow arrows in the Figure 3A and are flat disc-shaped. Non-activated ruptured platelets are shown by red arrows and are spherical. Therefore, we can observe that there are more activated platelets on the surface of PK group, while the surface of other three groups is mostly non-activated platelets. As shown in Figure 3A, platelets can adhere to almost any spot on the frosted PEEK surface, including ridges and gullies. However, on the surface of ST-PK and SA-PK groups, the platelet volume was much larger than the inner diameter of micropore and could not enter the inside of microstructure.

The amount of platelet adhesion on a single PEEK surface of each group was quantified using the LDH assay and the results are shown in Figure 3B. The number of platelets adhered to the surface of each PK group was 122.60 ± 32.50, which was the least of the four groups. The number of surface adherent platelets in each SP-PK group was 270.50 ± 70.75, which was significantly higher than that in the other three groups. The number of platelet adhesions on the surface of each ST-PK group and SA-PK group was 205.33 ± 20.66 and 201.13 ± 17.59, respectively, which was higher than that of the PK group and lower than that of the SP-PK group.

The results of PDGF-BB release from PRP coatings on different PEEK surfaces evaluated using Elisa kits are shown in Figure 3C. The release trend of PDGF-BB in the four groups was basically the same, and the release gradually decreased with the increase in time. At each time point accumulated from 0 h, the amount of PDGF-BB released was higher in the SP-PK group than in the other three groups. The amount of PDGF-BB released in the PK group was lower than that in the other three groups. Cumulative PDGF-BB release was consistently higher in the ST-PK group than in the SA-PK group until 4 h after which the cumulative release was reversed in both groups. In addition, as shown in Figure 1, the cumulative release of PDGF-BB at the beginning (0 h) and the end (24 h) were analyzed separately (Figures 3D, E). It can be found that the release of PDGF-BB in the SP-PK group was significantly higher than that in the other three groups at both time points, while no statistical difference was found among the other three groups.

### 3.3 *In vitro* cell-material interactions assay

#### 3.3.1 Cell adherence

**FIGURE 4:**
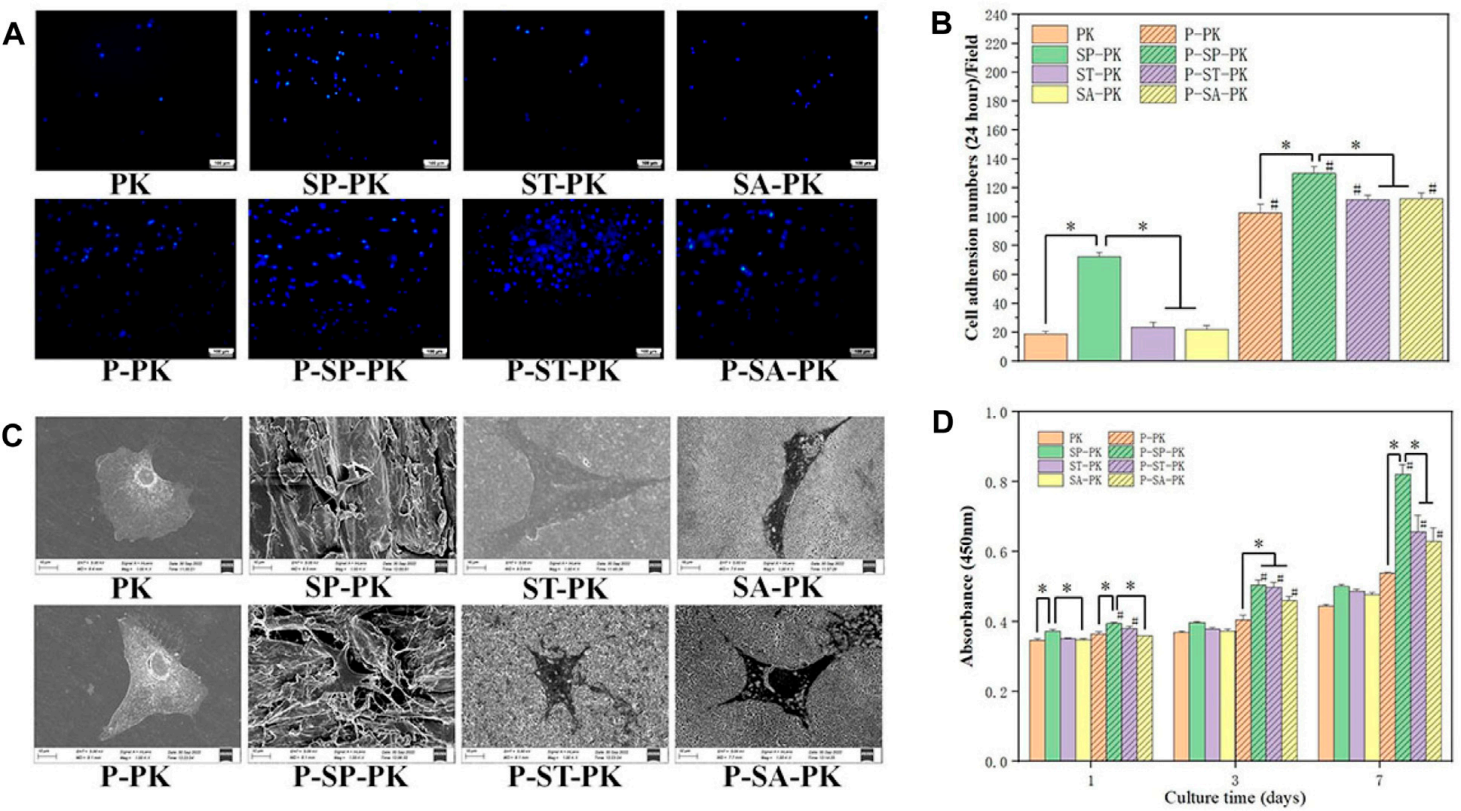
**(A)** DAPI staining of nuclei of adherent cells on the surface of PEEK samples from each group. **(B)** Quantitative analysis of the nuclei of adherent cells on the surface of each PEEK sample, *n* = 3. **(C)** SEM images of adherent cells on the surface of PEEK samples from each group. **(D)** CCK-8 results of the proliferation of MC-3T3-E1 cells on the PEEK surface of each group for 1,3, and 7 days, *n* = 3.* indicate *p* < 0.05, ^#^ represents that compared with the same treatment method, the group incubated with PRP was significantly higher than the group not incubated, *p* < 0.05.

After MC3T3-E1 cells were cultured on the surface of each PEEK sample for 24 h, nuclei were stained with DAPI and counted, as shown in Figure 4A. It was evident that the nuclei on the surface of SP-PK group were the densest either before or after the incubation of PRP. It can be clearly seen that for the PEEK surface treated in the same way, the density of nuclei on the PEEK surface after PRP incubation was significantly higher than that before incubation. The nuclei on the surface of the SP-PK group were the densest either before or after PRP incubation. The number of nuclei in the ST-PK and SA-PK groups was significantly higher than that in the PK group, but there was no significant difference between the two groups. The above results were confirmed by the cell counts on the PEEK surface of each group shown in Figure 4B.

The cells were cultured on the PEEK surface of each group for 24 h, and the cell adhesion morphology was observed using SEM (Figure 4C). The cells on the surface of the PK group without PRP incubation tended to be undifferentiated and round with few cell pseudopodia. The cells on the surface of the PK group incubated with PRP and the other three groups without PRP showed mild differentiation and obvious pseudopodia. The cells on the surface of the SP-PK group, especially after the surface was incubated with PRP, appeared more stereoscopic and formed more connections between cells. The cells and their synapses are too large to enter the microstructures of ST-PK (P-ST-PK) and SA-PK(P-SA-PK) groups, but the cells can “climb” along the microstructures of SP-PK(P-SP-PK) group.

#### 3.3.2 Cell proliferation

Figure 4D shows the results of cell proliferation using CCK-8 assay after culturing the cells on the surface of different samples in each group for 1,3, and 7 days. On the first day, the OD values of P-ST-PK and P-ST-PEEK groups were significantly higher than those of ST-PK and ST-PEEK groups, respectively. The OD values of cells on the surface of frosted PEEK were significantly higher than those in the other three groups, regardless of whether PRP was incubated or not. Meanwhile, the OD value of the P-ST-PK group was significantly higher than that of the P-SA-PK group. On the third day, the OD values of the P-SP-PK,P-ST-PK, and P-SA-PK groups were significantly higher than those of the SP-PK,ST-PK, and SA-PK groups. The P-PK group had a significantly lower OD value than the P-SP-PK,P-ST-PK, and P-SA-PK groups. On the seventh day, the P-SP-PK,P-ST-PK, and P-SA-PK groups showed significantly higher OD values than the SP-PK,ST-PK and SA-PK groups. The OD values of the P-SP-PK group were significantly higher than those of the other three groups after PRP incubation. These results indicated that surface incubation of PRP could improve cell proliferation ability of the modified PEEK surface. In this study, the frosted PEEK surface with PRP coating had the strongest ability to promote cell proliferation.

#### 3.3.3 Cell differentiation

**FIGURE 5:**
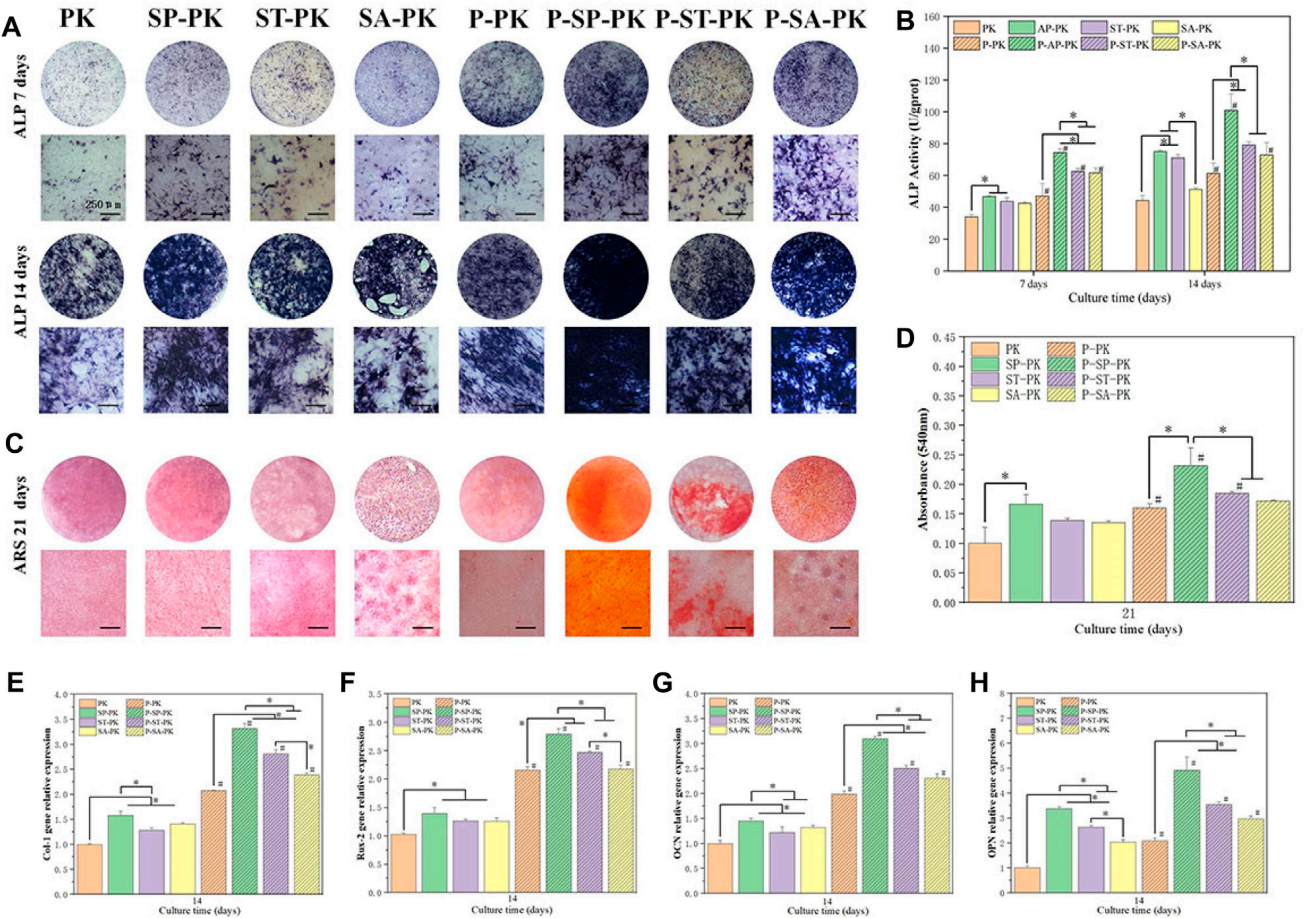
**(A)** ALP staining results of MC3T3-E1 cultured on PEEK surface for 7 days and 14 days in each group. **(B)** ALP quantitative analysis results of cells cultured on PEEK surface for 7 days and 14 days, *n* = 3. **(C)** ARS staining of cells cultured on PEEK surfaces for 21 days in each group. **(D)** Quantitative ARS analysis of cells cultured on PEEK surfaces for 21 days in each group, *n* = 3. **(E–H)** Osteogenesis-related gene (Col-1, Rux-2, OPN, OCN) expressions of MC3T3-E1 were evaluated by RT-qPCR (*n* = 3) 14 days after cultured on the surface of PEEK surface of each group, *n* = 3.* indicate *p* < 0.05, # represents that compared with the same treatment method, the group incubated with PRP was significantly higher than the group not incubated, *p* < 0.05.

The ALP activity is one of the commonly used indicators to evaluate osteogenic differentiation ability of cells. Therefore, we performed ALP staining and quantitative evaluation of cells on the surface of different groups of samples at specific time points, and the results are shown in Figures 5A, B. ALP staining results at the same time point showed that before PRP incubation, the color of PK group was the lightest, and the color of SP-PK group was the darkest. After incubation with PRP, the P-PK group showed the lightest staining and the P-SP-PK group showed the darkest staining. The staining of P-PK, P-SP-PK,P-ST-PK, and P-SA-PK groups was darker than that of PK, SP-PK,ST-PK, and SA-PK groups, respectively. These results tend to be consistent with the results of the quantitative analysis in Figure 2. The quantitative results on days 7 and 14 showed that the OD values of P-PK,P-SP-PK,P-ST-PK and P-SA-PK groups were higher than those of PK, SP and SA-PK groups without PRP incubation, respectively. The P-SP-PK group had a significantly higher OD value than the P-PK,P-ST-PK, and P-SA-PK groups. The OD values of the P-ST-PK and P-SA-PK groups were significantly higher than those of the P-PK group at 7 days. At 14 days, the P-ST-PK group had higher OD values than the P-PK group.

ARS staining and quantification represent an extracellular matrix calcium deposition in the late stages of osteogenic differentiation and the results are shown in Figures 5C, D. For APS staining, the trend between the groups was the same as for ALP staining described above. The P-SP-PK group had the most calcium deposition, which was bright red with large calcium nodules. The ARS quantitative analysis showed that the OD value of the SP-PK group was significantly higher than that of the PK group. The P-SP-PK group had significantly higher OD values than the other three groups incubated with PRP. The OD values of P-PK,P-SP-PK, and P-ST-PK groups were significantly higher than those of PK, SP and ST-PK groups, respectively.

Figures 5E–H show the expression levels of four osteogenic genes (Col-1,Rux-2,OPN,OCN) in surface cells of each group of samples. The results showed that the incubation of PRP significantly increased the expression level of osteogenic genes on different modified PEEK surfaces. The expression levels of osteogenic genes in the P-SP-PK group were significantly higher than those in the P-PK,P-ST-PK, and P-SA-PK groups. The expression levels of most osteogenic genes (Col-1,OPN,OCN) in P-ST-PK and P-SA-PK groups were significantly higher than those in P-PK group. There was no significant difference in the expression levels of most osteogenic genes between ST-PK and SA-PK groups, but the expression levels of half of the osteogenic genes in the P-ST-PK group were significantly higher than those in the P-SA-PK group. In the group without PRP incubation, the expression level of osteogenic genes in SP-PK group was the highest, and the expression level of osteogenic genes in SP-PK,ST-PK and SA-PK groups was significantly higher than that in PK group.

The above results indicate that PRP greatly enhances the ability of various PEEK surfaces to promote cell osteogenic differentiation. In this study, P-SP-PK was found to have the strongest ability to promote the osteogenic differentiation.

### 3.4 *In vivo* bone repair

#### 3.4.1 Microcomputed tomography (Micro-CT) evaluation

**FIGURE 6:**
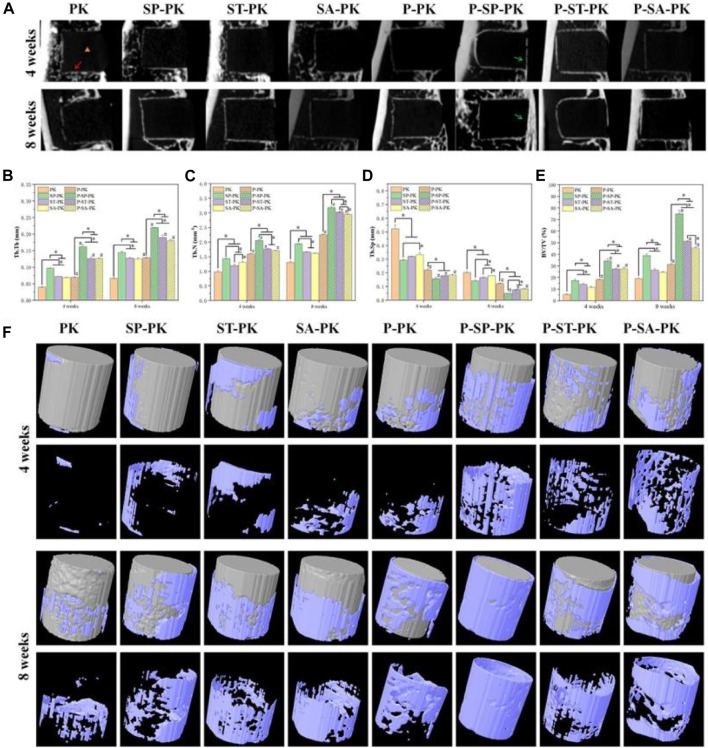
**(A)** 2D images of *in vivo* osteogenesis in each group of samples. Yellow triangle: PEEK material location; Red arrow: new bone; Green arrow: new bone at the tibial burr site. **(B–E)** Quantitative analysis of *in vivo* samples of each group for bone volume fraction (BV/TV), trabecular bone number (Tb. N), trabecular bone thickness (Tb. Th) and trabecular bone separation (Tb. Sp), *n* = 3. **(F)** 3D reconstruction of PEEK samples and surrounding new bone in each group. PEEK samples are shown in gray, and new bone is shown in blue. * indicate *p* < 0.05, # represents that compared with the same treatment method, the group incubated with PRP was significantly higher than the group not incubated, *p* < 0.05.

The SD rat tibial defect model is a commonly used animal model to evaluate the osteogenic ability of implants. At 4 and 6 weeks after operation, the fixed tibial specimens described in 2.6.1 were scanned by CT, three-dimensional reconstruction was performed, and the microstructure parameters of bone tissue were calculated. Using DATA Viewer processing, two-dimensional images were obtained, as shown in Figure 6A. The black rectangle where the orange triangle is located is the implanted PEEK material, and the white line shown by the red arrow is the new bone tissue around the material. At 4 weeks, new bone formation around the implants in the PK group was less and punctuated. In the ST-PK and SA-PK groups, the new bone formed a short continuous in the local area. The SP-PK group showed longer continuous new bone around the implant, and the new bone was thicker than the other three groups. At 4 weeks, the new bone around the implants in each PRP group roughly wrapped the implants in a U-shape, and the new bone in the P-SP-PK and P-ST-PK groups was significantly thicker than that in the other two groups. In the P-SP-PK group, the continuous new bone was also formed at the open end of the implant (green arrow). Meanwhile, the new bone formation around the implants in P-PK, P-SP-PK, P-ST-PK and P-SA-PK groups was more continuous and thicker than that in PK, SP-PK, ST-PK and SA-PK groups, respectively. At 8 weeks, the new bone mass around the implants in each group was consistent with that at 4 weeks: P-PK > PK, P-SP-PK > SP-PK, P-ST-PK > ST-PK, P-SA-PK > SA-PK, and at the same time: PK < ST-PK/SA-PK < SP-PK. P-PK < P-ST-PK/P-SA-PK < P-SP-PK.

Figure 6F shows the 3D reconstruction results of 4-week and 8-week tibial specimens from each group. The gray columns are the reconstructed PEEK implants of each group, and the new bone on the surface is blue-purple. The volume of new bone attached to the prosthesis surface in each group incubated with PRP was significantly higher than that in each group without incubated PRP. With the extension of time, the volume of new bone on the implant surface of each group at 8 weeks was significantly higher than that at 4 weeks. The volume of new bone around the implants was the largest in the P-SP-PK group at either 4 or 8 weeks.

The results of CT were further evaluated by quantitative analysis, including bone volume fraction (BV/TV), trabecular bone number (Tb. N), trabecular bone thickness (Tb. Th), and trabecular bone separation (Tb. Sp) (Figures 6B–E). The BV/TV, Tb. N and Tb. Th of each group incubated with PRP was significantly higher than that of each group incubated without PRP Among all the groups BV/TV, Tb. N and Tb. Th in the P-SP-PK group was significantly higher than that in the other groups. At the same time, the trend of the results of Tb. Sp is opposite to that described above.

#### 3.4.2 Histological evaluation

**FIGURE 7:**
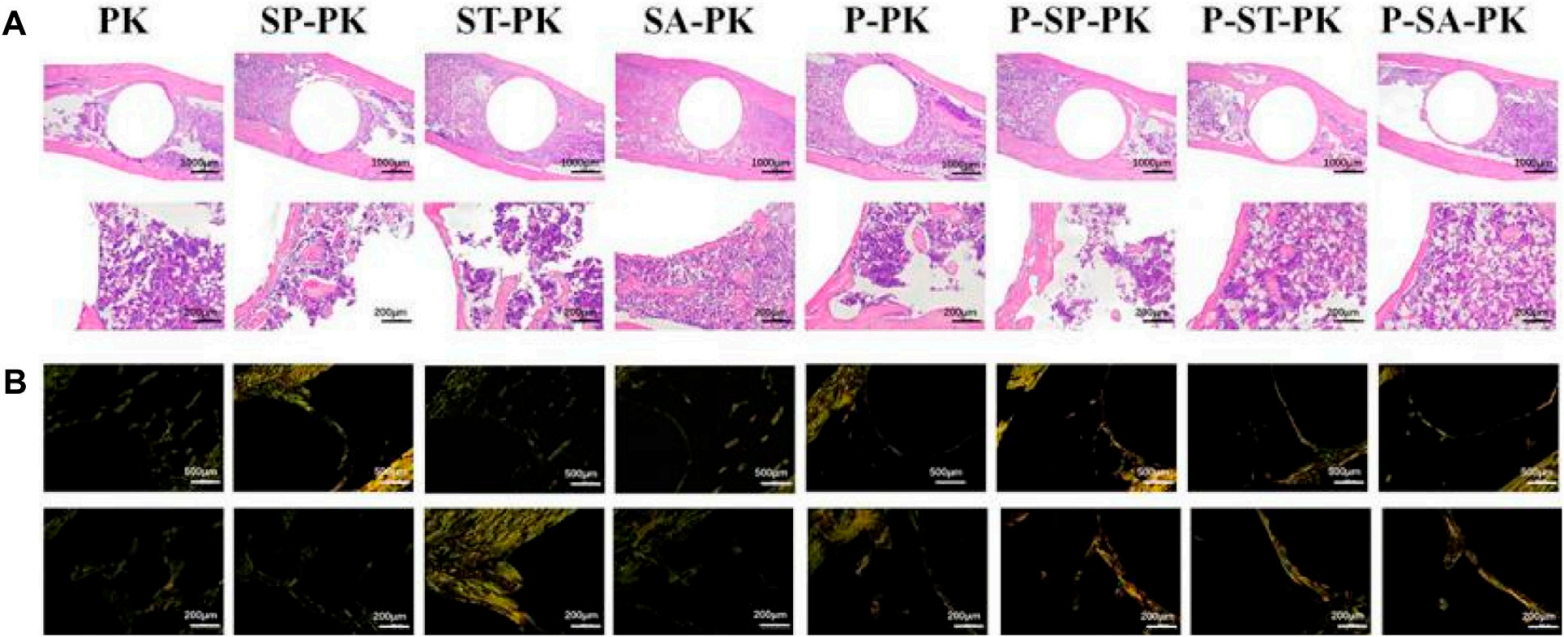
**(A)** The HE staining results of the tibial specimens of each group. **(B)** Sirius staining results of tibial specimens from each group; Orange represented typeⅠcollagen, green represented type III collagen.

The HE staining results of the tibial specimens at 8 weeks are shown in Figure 7A. The position of the black triangle is the space left after the removal of the implant, the red arrow shows the new bone, and the yellow triangle shows the medullary cavity and bone marrow tissue. It can be clearly seen that the pink ring formed by the bone tissue around the implant in the HE staining results of each group incubated with PRP was more complete and the wall was thicker than that of each group without incubation with PRP. The new bone around the modified PEEK implant was significantly more than that around the original PEEK. Meanwhile, the P-SP-PK group had the most new bone formation among all groups.

Figure 7B shows the Sirius staining results of tibial specimens from each group at 8 weeks, showing the distribution of different types of collagen. The groups without PRP incubation were dominated by green immature type III collagen. The green immature type III collagen was predominant in the groups without PRP incubation, whereas the mature orange type I collagen was predominant in the groups with PRP incubation. The P-SP-PK group showed the darkest orange among all groups.

**FIGURE 8:**
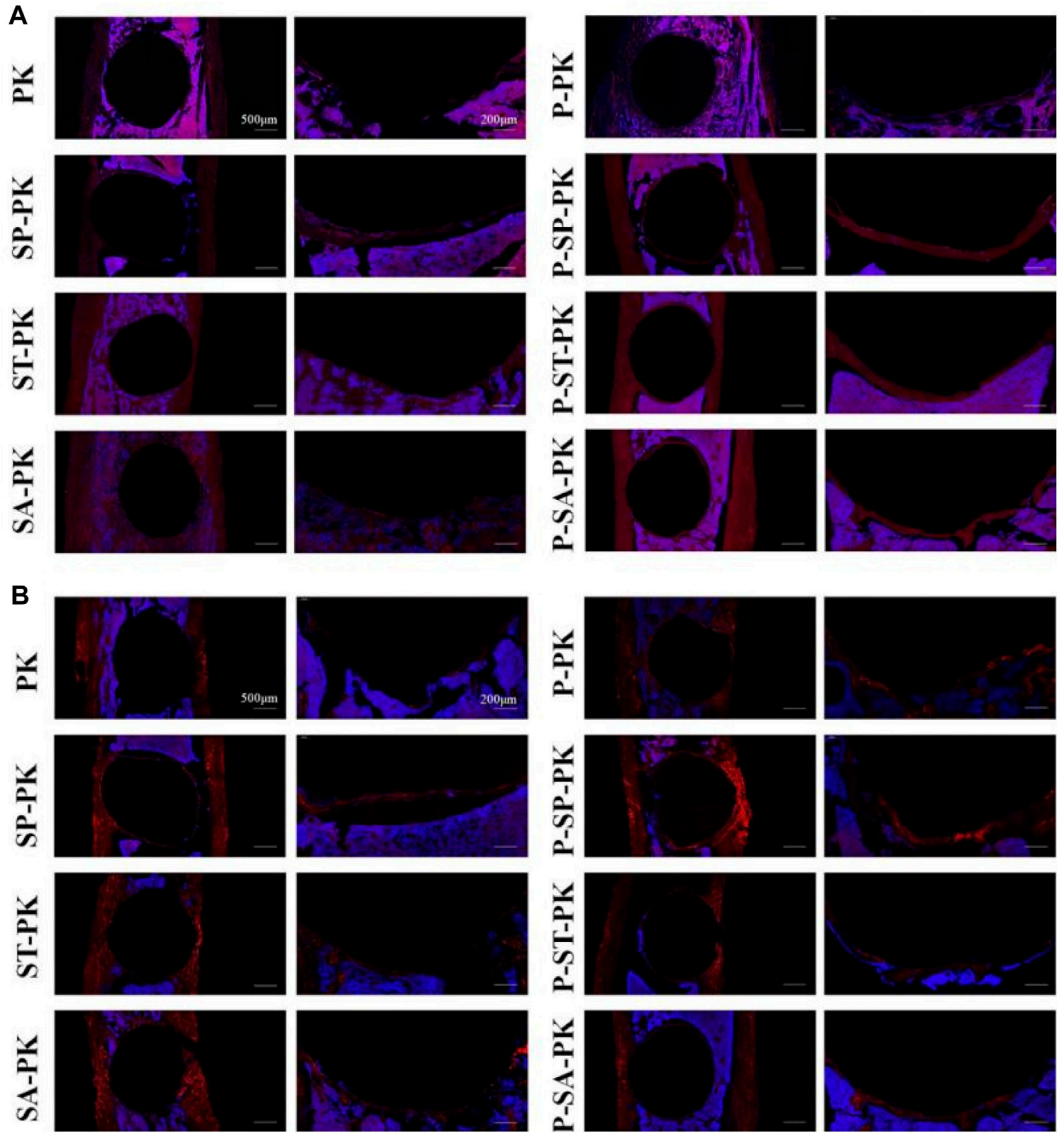
**(A)** OCN immunofluorescence staining of tibial samples from each group. Red represents the expression of OCN, and blue is the nucleus. **(B)** OPN immunofluorescence staining of tibial samples from each group. Red represents the expression of OPN, and blue is the nucleus.

Figure 8 show the immunofluorescence staining results of CON and OPN in tibial sample sections of each group at 8 weeks, respectively. In Figure 8A, the dark red represents the expression of OCN and the blue is the nucleus. In Figure 8B, the bright red represents OPN expression and the blue represents the nucleus. It could be seen that the expressions of OCN and OPN in the PRP incubated group were obviously higher than those in the non-incubated group, respectively:the red ring expression band was more complete and the wall was wider. The expression of OCN/OPN in each PEEK surface modification group was significantly higher than that in the original PEEK group, and the P-SP-PK group had the highest expression.

## 4 Discussion

In the process of bone defect repair, it is important to find suitable bone replacement materials. At present, bone replacement materials used in clinical practice mainly include metal, ceramic, and autologous (allogeneic) bone grafts. However, they all have limitations, such as high elastic modulus, large brittleness, and are difficult to obtain (HuiskesWeinans and Van, 1992).At present, bone replacement materials used in clinical practice mainly include metal, ceramic, and autologous (allogeneic) bone grafts. However, they all have limitations such as high elastic modulus, large brittleness, and difficulty in obtaining them (Kokubo et al., 2003). In recent years, PEEK has become one of the most promising bone replacement materials due to its mechanical strength and elastic modulus close to bone, chemical inertance, and radiation permeability. However, the high hydrophobicity and biological inertness of raw PEEK materials are not conducive to the adhesion, proliferation and differentiation of bone cells on its surface, which hinders its clinical application. Therefore, modification of PEEK materials to improve their biological activity has become a hot research topic (Gu et al., 2021; Dondani et al., 2023).

In this study, three methods were selected to modify the surface structure of PEEK, including sandpaper grinding, concentrated sulfuric acid sulfonation and sulfur trioxide gas fumigation. The three modification methods have their own characteristics. Sandpaper polishing is one of the simplest ways of PEEK surface modification, which can significantly improve the surface roughness of PEEK. Previous studies have shown that exfoliated PEEK surfaces can promote the proliferation and differentiation of rat bone marrow mesenchymal stem cells *in vitro*. In orthopedic clinics, hydroxyapatite coating that alters the surface roughness of metals is also one of the mature methods to improve the ability of joint prosthesis surface to integrate with the host bone (Søballe, 1993). Sulfuric acid can form 3-dimensional connected pore structure on PEEK surface through sulfation, thereby improving the bioactivity of PEEK surface. At the same time, these pore structures increase the ability of the PEEK surface to carry drugs and bioactive molecules, providing a basis for further improving the biological activity of PEEK. In our previous study, gaseous sulfur trioxide was used to fabricate a hydrophilic and porous PEEK surface. The study has shown that the surface has good mineralization ability and is conducive to cell adhesion, proliferation and differentiation (Wan et al., 2020).

According to the SEM picture results, it can be seen that the size of the microstructure produced by sandpaper polishing on the PEEK surface is much larger than that of the other two methods. We believe that this difference in microstructure size is one of the reasons why the polished sandpaper surface can carry PRP much more than the other two. EDS results showed that the content of S element on the surface of the ST-PK group was much higher than that of the other three groups. Combined with the FTIR results, we speculate that the S element is in the form of -SO_3_H on the surface of PEEK materials in ST-PK group. Previous studies have shown that sulfonation of PEEK in concentrated sulfuric acid is accompanied by mild dissolution of PEEK materials (Ma et al., 2020). However, according to our observation, in the reaction between gaseous sulfur trioxide and PEEK, a darker and darker yellow coating will be formed on PEEK surface over time. We believe that this coating protects PEEK to some extent from further corrosion by gaseous sulfur trioxide while also ensuring adequate reaction of the same layer of PEEK surface with sulfur trioxide. Thus, the micropores on the PEEK surface of the ST-PK group were less dense than those of the SA-PK group, as observed in the SEM images. At the same time, the more adequate reaction of the PEEK surface with gaseous sulfur trioxide made the PEEK surface of the ST-PK group contain more -SO_3_H than that of the SA-PK group, and therefore have more S elements. The abrasive surface of PEEK material reduces the hydrophilicity of PEEK material, which is consistent with the results of previous studies (Mahjoubi et al., 2017). There are many factors affecting the hydrophilicity of materials, including surface structure and surface group properties Even though the ST-PK group sample has increased roughness compared with the original PEEK material, the introduction of large amount of -SO3H still increases its hydrophilicity (Wang et al., 2016). However, for SA-PK group, the surface microstructure was still the main reason for the weakening of hydrophilicity because the amount of -SO3H introduced into the surface was too small.

Whether used as bone or dental implants, sufficient mechanical strength is a prerequisite for implant materials, especially compressive resistance (Koh et al., 2019). Therefore, we examined the compression resistance of different samples. Not surprisingly, the sulfur trioxe treated PEEK exhibits the closest compressive resistance to the original PEEK. This may be related to the protective ability of yellow colloidal coating formed on the PEEK surface during the reaction of PEEK with gaseous sulfur trioxide as we mentioned above. It is this yellow coating that prevents excessive dissolution of PEEK material. However, unexpectedly, the SP-PK group, which had a significantly larger surface microstructure under electron microscopy, performed better in the compressive stress test than the SA-PK group. This may be due to the rapid dissolution of PEEK in concentrated sulfuric acid. On the other hand, we used AFM to observe the finer structure of different groups of sample surfaces. The nanoscale surface roughness of SA-PK group was significantly higher than that of the other three groups, which also confirmed that the corrosion of PEEK material by concentrated sulfuric acid was significantly higher than that of the other groups, which was also the reason for the worst compressive resistance of SA-PK group.

PRP is rich in various growth factors which can promote the proliferation and differentiation of various cells. It has been widely used in orthopedics, cardiothoracic surgery, plastic surgery, dermatology, dentistry, and diabetic wound healing (Everts et al., 2021; Mijiritsky et al., 2021; Oneto and Etulain, 2021). In addition, several studies have reported that PRP has an immunomodulatory effect, which can promote the polarization of macrophages to anti-inflammatory M2, while inhibiting the polarization of macrophages to pro-inflammatory M1 (Jiang et al., 2021). Studies have shown that implants can trigger an inflammatory response after being implanted in the body, and excessive inflammatory response is not conducive to the integration of implants with the host (Brown and Badylak, 2013; Klopfleisch and Jung, 2017). At the same time, wear particles produced after joint prosthesis implantation will trigger macrophage M1 polarization, which will lead to osteolysis and ultimately implant failure (Gao et al., 2018; Guo et al., 2022; Lu et al., 2023). All these results indicate that PRP is a potentially ideal bioactive coating on PEEK surface. However, most of the existing studies are on doping of PRP with other degradable materials. Therefore, this study can be regarded as a preliminary attempt to apply PRP on a PEEK surface alone. Although previous studies have shown that exogenous growth factors on the surface of PEEK have a good osteopromoting effect, PRP still has two major advantages: autologous and relatively low cost. In other words, the autologous PRP coating on the PEEK surface will not cause immune rejection; the mature PRP preparation technology reduces the threshold for promotion.

The electron microscope images of PRP adhered to the PEEK surface of each group showed that the platelets on the surface of the samples in P-SP-PK,P-ST-PK and P-SA-PK groups were mostly inactive and spherical (Goodman, 1999), which was beneficial to the preservation of platelets and the release of more growth factors after implantation *in vivo*. Although PRP can be activated in many ways, such as repeated freezing and thawing, the use of activators, etc. In this study, we did not use any activation method to activate PRP *in vitro*. The main reasons are as follows: 1. The use of *in vitro* activation can produce a strong platelet activation effect, which may affect our judgment on the effect of PEEK morphology on platelet status and growth factor release; 2. Activated platelets release a large amount of growth factors in a short period of time, and exposure of growth factors to PEEK surface for too long may affect their biological activity; 3. There is a large amount of collagen in the extracellular matrix and the body, and studies have shown that collagen itself is a good activator of PRP (Hechtman et al., 2011; Zhang et al., 2019). At the same time, the large surface microstructure of SP-PK is most conducive to the capture of platelets. This is also the reason why the samples in PRP-SP-PK group released the most PDGF-BB. It should be noted that the amount of PDGF-BB released at 0 was not zero in each group. This indicated that the PRP solution itself contained PDGF-BB and was adsorbed on the surface of samples in each group. At the same time, we believe that the large size of the microstructure on the surface of SP-PK expanded the adsorption area and was conducive to the adsorption of PDGF-BB, which was the reason why the samples in SP-PK group adsorbed the most PDGF-BB at 0 h. Hydrophilicity is one of the factors affecting cell adhesion on the surface of materials, and hydrophilic materials are more conducive to cell adhesion. It is obvious that the three PEEK surface modification methods in this study all changed the water contact Angle of PEEK material to varying degrees. However, the most hydrophobic group of SP-PK samples had the most platelets adhering to their surfaces. We speculated that in this study, the surface structure of PEEK was still the main influencing factor in the process of PRP incubation.

We used DAPI to stain the nuclei of MC3T3-E1 on the surface of each group; platelets did not have nuclei and could not be stained. The number of nuclei on the surface of the samples in each group incubated with PRP was significantly higher than that in the non-incubated groups. This indicates that PRP coating significantly improves the cell affinity of PEEK surface and facilitates cell adhesion. At the same time, electron microscope images showed that the number of cell pseudopodia on the surface of each group of samples incubated with PRP was more, which also confirmed that PRP coating improved the cell adhesion ability of PEEK material (Liu et al., 2018; Wan et al., 2019). In the SEM images, the cells were embedded in the surface microstructure of the sanded PEEK, indicating that the larger microstructure can be beneficial to the capture of cells, which is also the reason why the number of cell adhesion in the SP-PK group is much more than that in the PK, ST-PK and SA-PK groups. For each group incubated with PRP, the number of platelets and growth factors incubated on the surface was the determinant of the number of adherent MC3T3-E1 cells. In addition, SEM images showed that the cells in PK, ST-PK and SA-PK groups spread better than those in SP-PK group, indicating that the flat surface of the material was more conducive to cell spreading. However, in practical clinical applications, the surface of materials such as SP-PK group that can incorporate cells can better achieve bone ingrosis, thereby enhancing the bonding force between the material and bone (Kuboki et al., 2001).

PRP is enriched with various growth factors, including VEGF, TGF, and PDGF. Previous studies have shown that these growth factors promote osteocyte proliferation (Wu et al., 2016; Hu and Olsen, 2017; Wang et al., 2023). Therefore, in the CCK-8 test, cell proliferation on the surface of the samples in each group incubated with PRP was significantly higher than that in the non-incubated group. Because growth factors promoted cell proliferation in a dose-dependent manner, the P-SP-PK group samples loaded with the most PRP had the strongest ability to promote cell proliferation. ALP is an important mineralization enzyme in the process of bone formation, metabolism, and regeneration. ALP activity is a marker activity in the early differentiation stage of osteoblasts, and it is a characteristic index to evaluate the differentiation of osteoblasts (Bai et al., 2020). Calcified nodules are another important indicator for osteoblast identification under *in vitro* culture conditions. When cultured *in vitro*, osteoblasts can not only show cell proliferation, differentiation, and matrix secretion but also form mineralized nodules similar to embryonic bone or woven bone *in vivo*, which is the last stage of osteoblast differentiation. Alizarin red staining is a specific staining for calcified nodules, which can directly reflect the mineralization ability of osteoblasts. In the ALP and ARS tests in this study, the groups incubated with PRP showed better ability to promote osteogenic differentiation than the groups without PRP. Numerous studies have confirmed that PRP can promote bone defects and has been widely used in clinical practice. However, the specific mechanism by which PRP promotes bone defect repair is still not fully understood. Although PDGF is a recombinant growth factor approved by the FDA for clinical use to promote bone regeneration, recent studies have shown that PDGF-BB alone does not significantly directly promote bone defect repair (Luvizuto et al., 2016; de Oliveira et al., 2017; Bai et al., 2020). However, studies have shown that PDGF-BB contributes to osteocyte proliferation and intraosseous angiogenesis (Wang et al., 2023). Studies have shown that TGF-β can promote the proliferation, chemotaxis, and early differentiation of osteoprogenitor cells through the Smad pathway and inhibit osteoclast differentiation by downregulating the RANKL/OPG secretion ratio (Wu et al., 2016). VEGF also plays an important role in bone defect repair. When the bone defect appears, blood vessels first invade the defect site, and then osteoprogenitor cells migrate to the defect to start the process of bone defect repair. The main role of VEGF is to promote angiogenesis and the integration of bone and neovascularization (Hu and Olsen, 2017; Grosso et al., 2023). Finally, in the RT-PCR assay, we confirmed that PRP coating on the PEEK surface significantly increased the expression of osteogenesis-related genes.

Next, to verify the osteogenic ability of the samples in each group *in vivo*, we established an SD rat tibial defect model. Tibia samples were collected at 4 and 8 weeks. In the 2D and 3D reconstructed CT images, it was obvious that the amount of new bone formation in the samples with PRP coating was significantly higher than that in the samples without PRP coating. The P-SP-PK group incubated with the most PRP had the most new bone formation. Subsequent quantitative analysis confirmed the above results. To further confirm the molecular mechanism of osteogenesis promoted by PRP coating on the PEEK surface, we performed HE, Sirius red, and immunofluorescence staining on tibial specimens. HE stain confirmed the formation of more complete and wider new bone around PEEK samples with PRP coating in each group. Sirius staining is a method to distinguish the type of collagen. Bone contains up to 90% type I collagen, while bone containing more type III collagen represents the immature state (Adel-Khattab et al., 2020; Metwally et al., 2020). Therefore, in this study, the newly formed bone on the surface of the samples in each group with PRP coating, especially in the P-SP-PK group, was more mature than that in the groups without PRP coating. Osteopontin (OPN) is secreted by osteoblasts and stored in the bone matrix. It can induce the maturation of mineralized bone matrix, regulate the formation of hydroxyapatite and bone salt deposition (Hou et al., 2022). Osteocalcin (OCN) appears in the late stage of osteoblast differentiation and has the function of regulating calcium homeostasis (Karsenty and Ferron, 2012). Compared with the groups without PRP coating, the expressions of OPN and OCN in the groups with PRP coating were significantly increased, which confirmed the osteogenic ability of PRP coating. Similarly, the P-SP-PK group expressed the most OPN and OCN.

However, this study still has some limitations. Firstly the release time of growth factors in PRP coating in this study was short. Second, although the PRP extraction procedure is mature, the loading efficiency of PRP is low when preparing native PRP layer on the PEEK surface. Finally, the immunoregulation and angiogenesis ability of the PRP coating on PEEK were not investigated in this study. In the future study, we plan to develop a PRP gel coating on PEEK surface to enable the sustained release of growth factors. The immunomodulatory and angiogenic effects of PRP coating on PEEK were also investigated.

## 5 Conclusion

In summary, three modifications were carried out on PEEK, including sandpaper grinding, gaseous sulfur trioxide fumigation and sulfuric acid sulfonation, and PRP coating was incubated on the PEEK surfaces. Among the three PEEK modified surfaces, the frosted surface was considered to be the most suitable surface for the preparation of PRP coating due to the most PRP carrying. *In vitro* and *in vivo* experiments, the PRP coating on the PEEK surface significantly improved its cell affinity, greatly promoted cell proliferation and differentiation, and finally achieved the ability to promote bone defect repair. All three modified surfaces enhanced the ability of PEEK to carry PRP, thereby further enhancing osteogenesis. Among them, the sandpaper surface was equipped with the most PRP and had the strongest osteogenic ability.

## Data Availability

The raw data supporting the conclusions of this article will be made available by the authors, without undue reservation.
